# Evaluating metal-organic precursors for focused ion beam-induced deposition through solid-layer decomposition analysis

**DOI:** 10.3762/bjnano.16.135

**Published:** 2025-11-04

**Authors:** Benedykt R Jany, Katarzyna Madajska, Aleksandra Butrymowicz-Kubiak, Franciszek Krok, Iwona B Szymańska

**Affiliations:** 1 Marian Smoluchowski Institute of Physics, Faculty of Physics, Astronomy and Applied Computer Science, Jagiellonian University, Lojasiewicza 11, 30348 Krakow, Poland; 2 Faculty of Chemistry, Nicolaus Copernicus University in Toruń, Gagarina 7, 87-100 Toruń, Poland

**Keywords:** backscattered electrons (BSE), carboxylates, energy-dispersive X-ray spectroscopy (EDX), focused ion beam (FIB), focused ion beam-induced deposition (FIBID), machine learning, scanning electron microscopy (SEM)

## Abstract

The development of modern metal deposition techniques like focused ion/electron beam-induced deposition (FIBID/FEBID) relies heavily on the availability of metal-organic precursors of particular properties. To create a new precursor, extensive testing using specialized gas injection systems is required along with time-consuming and costly chemical analysis typically conducted using scanning electron microscopy (SEM). This process can be quite challenging due to its complexity and expense. Here, the response of new metal-organic precursors, in the form of supported thick layers, to the ion beam irradiation is studied through analysis of the chemical composition and morphology of the resulting structures. This is done using SEM backscattered electron/energy-dispersive X-ray spectroscopy along with machine learning data processing techniques. This approach enables a comprehensive fast examination of precursor decomposition processes during FIB irradiation and provides valuable insights into how the precursor’s composition influences the final properties of the metal-rich deposits. Although solid-layer irradiation differs from gas-phase deposition, we think that our method can be employed to optimize pre-screen and score new potential precursors for FIB applications by significantly reducing the time required and conserving valuable resources.

## Introduction

A variety of nanomanufacturing techniques, such as optical and electron-beam lithography, nanoimprint lithography, atomic layer deposition, chemical mechanical polishing, and laser nanopatterning, enable the creation of nanostructures and nanoscale devices. However, a major limitation of these methods is their inability to effectively produce high-resolution three-dimensional nanostructures [1–4]. In contrast, focused electron or ion beam-induced deposition (FEBID/FIBID) allows for the precise fabrication of two- and three-dimensional nanostructures with well-defined shapes and dimensions ranging from 5 to 10 nm [1,5–10]. This high spatial resolution is achieved by precisely controlling the position and duration of the electron or ion beam pulses. In FEBID and FIBID, volatile precursor molecules are delivered to the substrate surface via a gas injection system (GIS), where they adsorb and are subsequently decomposed by a focused electron or ion beam with energies in the kiloelectronvolt range. While commercial FIBID systems commonly employ Ga^+^ ions, alternative ion species such as He^+^, Ne^+^, Ar^+^, or Xe^+^ can also be used [11–14]. The resulting FEBID/FIBID deposits are widely used for repairing photolithographic masks and printing or modifying integrated circuits. In addition, they are applied for the fabrication or modification of cantilevers in AFM and scanning optical near-field microscopy, and as plasmonic materials [15–19]. FEBID/FIBID techniques combine the advantages of direct-write lithographic processes, for example, high spatial resolution, site-specificity, no need for masks, and resistance, with the flexibility of depositing materials on non-planar surfaces [4–5,14].

The FIBID method has several advantages compared to the FEBID technique in depositing thin films on substrates. First, ions generate more secondary electrons on the substrate surface than electrons, leading to faster deposition growth (around 100 times). Second, FIBID deposits have higher metal content and lower resistivity compared to FEBID. However, there are some disadvantages to FIBID, such as the larger size of noble gas and metal ions that penetrate to smaller depths in solids and result in significant beam-induced substrate defects (e.g., Ga atom implantation). Additionally, material growth is required to compete with the FIB milling process [4,9]. The use of ions instead of electrons, like in FEBID, offers several benefits, including enhanced film quality and adhesion, better control over the growth process, and greater flexibility in material selection (the ability to deposit a variety of different materials). The usage of ions opens new possibilities for materials development and applications [20–21].

Until now, the development of FEBID has relied on precursors used for chemical vapor deposition (CVD), a thermally driven process. However, these kinds of precursors were not optimized for the electron- and ion-driven FEBID and FIBID processes [4–5,14]. Important classes of FEBID-tested compounds for group-11 elements have been β-diketonates and carboxylates. These compounds were used previously in CVD, and β-diketonates are the most common CVD precursors, yielding films of high purity up to 99 atom % [5,15,22–26]. In FEBID, silver(I) carboxylates, in contrast to β-diketonates, result in high metal content in the deposits. Recent research [5] using [Ag_2_(μ-O_2_CR)_2_], where R = CF_3_, C_2_F_5_, C_3_F_7_, *t*-Bu, or C(Me)_2_Et, showed that these carboxylates can be dissociated via focused electron beams, yielding deposits with satisfying metal content (purity up to 76 atom % Ag). However, for the copper(II) carboxylate [Cu_2_(μ-O_2_CC_2_F_5_)_4_], the fabricated materials have only up to 23 Cu atom % [5,15,22–26]. This shows that the electron beam-induced decomposition is influenced by the ligand and also by the coordination center.

Due to the key role played by secondary electrons in the decomposition of FIBID precursors, FIBID precursor compounds are limited to those used and tested in FEBID processes. Preliminary studies of new or potential FEBID precursors employ electron ionization mass spectrometry and gas-phase cross-beam experiments (dissociative ionization and dissociative electron attachment), but more informative are investigations into the interactions of molecules adsorbed on the surface, such as electron-stimulated desorption, high-resolution electron energy loss spectroscopy, and focused electron beam secondary ion mass spectrometry [5].

To characterize compounds in terms of their applicability in the FIBID process, comparisons are made with the FEBID process, and decomposition mechanisms are proposed. Studies have been conducted on ion–molecule interactions in both solid and gas phases. Ultrahigh vacuum experiments on a few monolayers of FIBID precursors, such as [Ru(CO)_4_I_2_], [(η^5^-C_5_H_5_)Fe(CO)_2_Re(CO)_5_], and [Fe(CO)_5_], have been used to elucidate decomposition pathways. These studies also enabled a clear distinction between the processes occurring under ion irradiation and under electron irradiation. Furthermore, investigations on the gold complex [AuMe_2_(hfac)] have allowed for the assessment of the influence of ion type (mass) and ion energy on molecular decomposition. Gas-phase interactions between [Fe(CO)_5_] molecules and ions of helium, neon, argon, and krypton were carried out. However, the authors noted that these results may not accurately reflect the behavior of the precursor on the surface during actual FIBID processing since the conditions under which the study was conducted (pressure and cleanliness) differ significantly from those of a typical FIBID process [14].

To guide the development of more effective precursors for FEBID, a pre-screening strategy combining electron ionization mass spectrometry (EIMS) and volatility testing is typically applied. EIMS reveals how ligands respond to electron irradiation; simple molecules such as CO and CO_2_ detach cleanly, making them “favorable” in FEBID. In contrast, anionic or polyhapto ligands, such as cyclopentadienyl (Cp) or allyl fragments, readily fragment form C*_x_*H*_y_* matrices, leading to film contamination and degradation of structural quality. Halogen atoms (e.g., Cl and Br) remain bound during initial irradiation and are typically released only at high electron doses, posing risks of side reactions or inhomogeneous film formation. Volatility is assessed via the sublimation temperature; precursors with an onset below 100 °C are considered suitable for efficient transport through a GIS. Those with higher sublimation points are unlikely to reach the deposition zone and are thus disqualified early. Despite its advantages, EIMS pre-screening has significant limitations; it cannot determine the final film composition, metal oxidation states, or actual deposition efficiency. Results are also sensitive to sample purity and ionization conditions, and the method does not replicate the real gas-phase or ion-beam environments encountered in FIBID [27].

One has to mention that testing new metal-organic precursors for the use in FEBID/FIBID is a tedious time-consuming task, which requires costly experimental (non-commercial) GIS systems. The primary objective of this precursor testing is to optimize deposition parameters, specifically targeting high metal content (favoring minimal impurities of gallium from the FIB source) and reduced ion currents. It is also important to optimize beam energy for the deposition. Tripathi et al. [27] correlated ion beam parameters to the deposition characteristics using Ga FIB and various available precursors. Several tests of many different new precursors have to be done before deciding which compound is the most promising one. Therefore, we used in our studies copper(II) and silver(I) carboxylate complexes such as non-fluorinated pivalate [Cu_2_(μ-O_2_C*t*-Bu)_4_]*_n_* [28–29], perfluorinated pentafluoropropionates [Cu_2_(µ-O_2_CC_2_F_5_)_4_] [30], [Ag_2_(μ-O_2_C_2_F_5_)_2_] [31] and the heteroligand complex with the same carboxylate and pentafluoropropamidine [Cu_2_(NH_2_(NH=)CC_2_F_5_)_2_(µ-O_2_CC_2_F_5_)_4_] [32], as new potential precursors for the applications in focus ion beam induced deposition (FIBID) using gallium ions. We focused on a commonly used 30 keV FIB ion beam energy in our systematic decomposition studies.

Here, we present a pathway for unraveling how the chemical composition of metal-organic precursors affects their decomposition when irradiated with FIB in the form of supported layers. A decomposition process of the studied layer was quantitatively monitored by scanning electron microscopy backscattered electron (SEM BSE) analysis. For each studied precursor, an optimal ion fluence was determined, defined as the ion fluence at which the sputtering of the formed metal-rich structures becomes the dominant process, exceeding the rate of precursor decomposition and material buildup. While sputtering occurs throughout ion irradiation, this point marks the transition beyond which further irradiation leads primarily to material removal rather than the structure growth. The structures formed at “the optimal” ion fluence were examined by scanning electron microscopy energy-dispersive X-ray spectroscopy (SEM EDX) together with machine learning-based hyperspectral data processing, which uses non-negative matrix factorization (NMF) to separate the EDX signals of structures from the ones of the substrate. As already shown, this type of analysis greatly enhances the applicability of SEM EDX for the analysis of nanostructures [33]. Finally, we determined the quantitative chemical composition of the formed metal-rich deposits (structures). While the decomposition of precursor layers presents a significant challenge compared to gas-phase deposition driven by differing mechanisms, our approach of layer analysis offers crucial insights into the fundamental physics of metal deposition from metal-organic precursors. Furthermore, we believe that our methodology could be effectively utilized as a valuable tool for precursor screening. The use of a new compound as a FIBID/FEBID precursor necessitates a series of preliminary tests to confirm volatility and sensitivity to secondary electrons. Refining the conditions for efficient precursor delivery via the GIS system and its subsequent decomposition under ion beam influence requires testing in a difficult-to-access experimental reactor. Given the time- and cost-intensive nature of analyzing the morphology and composition of the deposits, we propose a method/approach to minimize studies within the experimental reactor and identify promising potential precursors.

## Testing Pathway and Methods Used

The proposed approach for effectively testing new metal-organic precursors involves a series of steps that are crucial to ensure accurate and comprehensive results. These stages include: (1) Deposition of the precursor onto a Si(111) substrate through sublimation using previously established parameters [22,29,32,34]. This step allows for precise control of the thickness of the precursor layer on the substrate. (2) Performing SEM imaging of the growth layers, which provide detailed information about the surface structure and composition of the precursor layer. They are essential for understanding how the precursors are decomposed under the following FIB irradiation and what kind of morphology is developed for the finally formed metal-rich structures. (3) Analyzing BSE images of the evolving surface morphology at successive stages of FIB irradiation in order to determine “the sputtering point”, that is, the threshold ion fluence at which the sputtering becomes the dominant process over structure growth, leading to the erosion of the formed metal-rich deposits. This threshold provides insight into the precursor’s resistance to ion bombardment and is crucial for assessing its stability and reactivity under processing conditions. (4) Collecting SEM EDX hyperspectral data, which involves acquiring multiple X-ray spectra from different points of the final sample morphology. This step allows for a more detailed analysis of the chemical composition and distribution of elements within the irradiated sample. (5) Decomposing SEM EDX hyperspectral data using advanced algorithms to separate and identify individual components within the irradiated sample. This process is essential for obtaining accurate and reliable information about the chemical composition of developed deposits. (6) Determining the chemical composition of the developed structures using the EDX ZAF technique, which is a high-resolution analytical method that can provide elemental information at the nanoscale. This step ensures precise identification and quantification of all elements present in the grown structures. (7) The final step involves examining the chemical composition of the resulted precursor layers and coupling it with ion beam parameters to score the precursor usability. This stage is crucial for determining the potential applications and limitations of new metal-organic precursors in various fields.

In the following, each of these steps will be explored in greater detail, providing a more in-depth understanding of the proposed pathway for successful testing of the new potential FIBID precursors.

## The Fabrication of the Precursor Thin Layer

**Figure 1:**
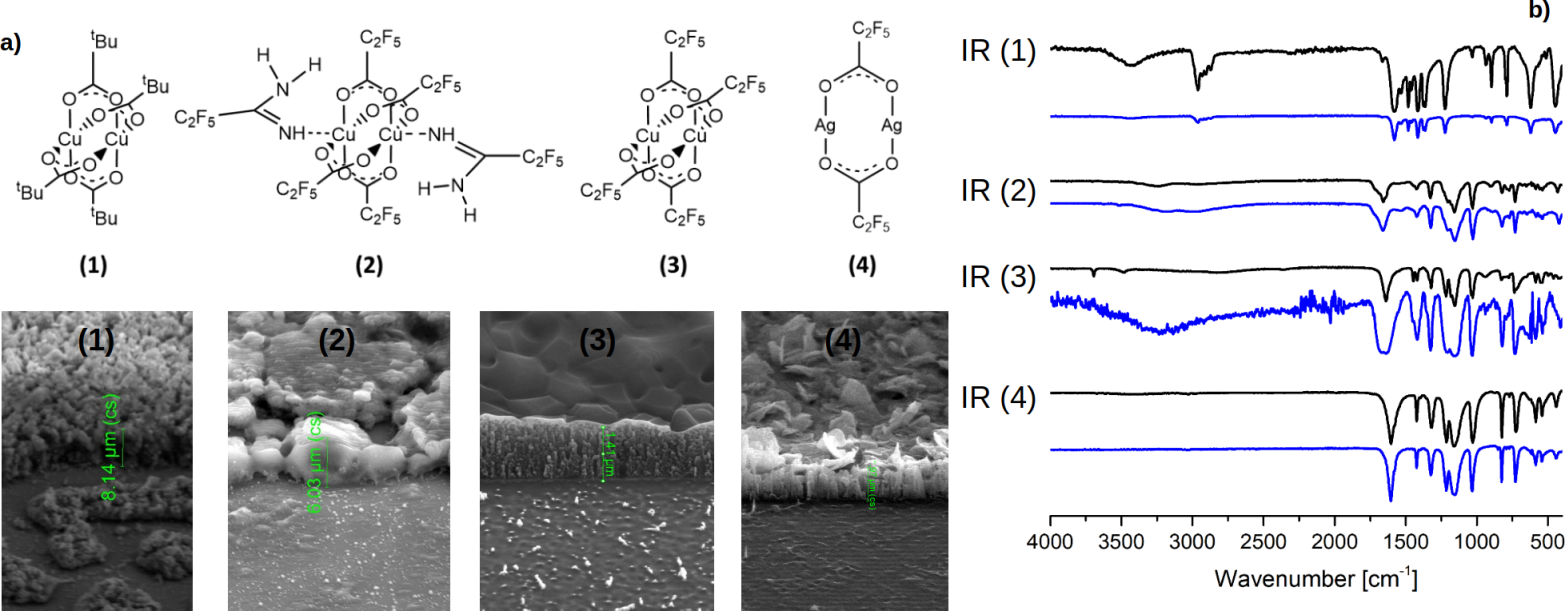
(a) SEM secondary electron images showing thickness and morphology of the precursor layers of **1**–**4**. (b) Infrared spectra before (black) and after sublimation on a silicon wafer (blue) for the compounds **1**–**4** (*p* = 10^−2^ mbar).

The metal-organic precursors films for the FIB/SEM experiments were deposited by sublimation using a glassware sublimation apparatus. The Si(111) wafer was placed in the special holder on the cold finger of the apparatus. The process was performed under a pressure of 10^−2^ mbar and at the following temperatures: 418 K − [Cu_2_(μ-O_2_C*t*-Bu)_4_] [29] (**1**), 393 K − [Cu_2_(NH_2_(NH=)CC_2_F_5_)_2_(µ-O_2_CC_2_F_5_)_4_] [32] (**2**), 413 K − [Cu_2_(µ-O_2_CC_2_F_5_)_4_] (**3**), and 413 K [Ag_2_(μ–O_2_CC_2_F_5_)_2_] (**4**) (Figure 1). The conditions for depositing layers of compounds **1** and **2** were previously determined [29,32]. In a similar way, the layers of the complexes **3** and **4** were prepared. The grown layer compositions were checked by IR spectroscopy before electron beam irradiation. IR spectra were registered with a Vertex 70V spectrometer (Bruker Optik, Leipzig, Germany) using a single reflection diamond ATR unit (400–4000 cm^−1^). IR spectra of the obtained layers and the initial compounds **1**–**4** are presented in Figure 1b. The spectra showed characteristic ν_as_(COO) and ν_s_(COO) bands of bonded carboxylate ligands (compounds **1**–**4**), as well as ν_as_(NH_2_), ν(=NH), δ(NH_2_), and ν(N=C−N) bands of coordinated amidine ligands for complex **2** (Figure 1b and Supporting Information File 1, Table S1) confirming the formation of suitable layers of the studied complexes.

SEM operating in secondary electron mode provided clear and precise images of the deposited layers (Figure 1a). The thickness of the grown layers was determined, yielding 8.14 µm for **1**, 6.03 µm for **2**, 1.41 µm **3**, and 1.07 µm for **4**. The experiments were conducted using a dual-beam SEM/FIB microscope “Quanta 3D FEG” manufactured by FEI. The microscope is equipped with a gallium FIB and an EDAX Ametek SDD EDX detector setup.

## FIB/SEM Irradiation Experiments and EDX Chemical Composition Quantification

**Figure 2:**
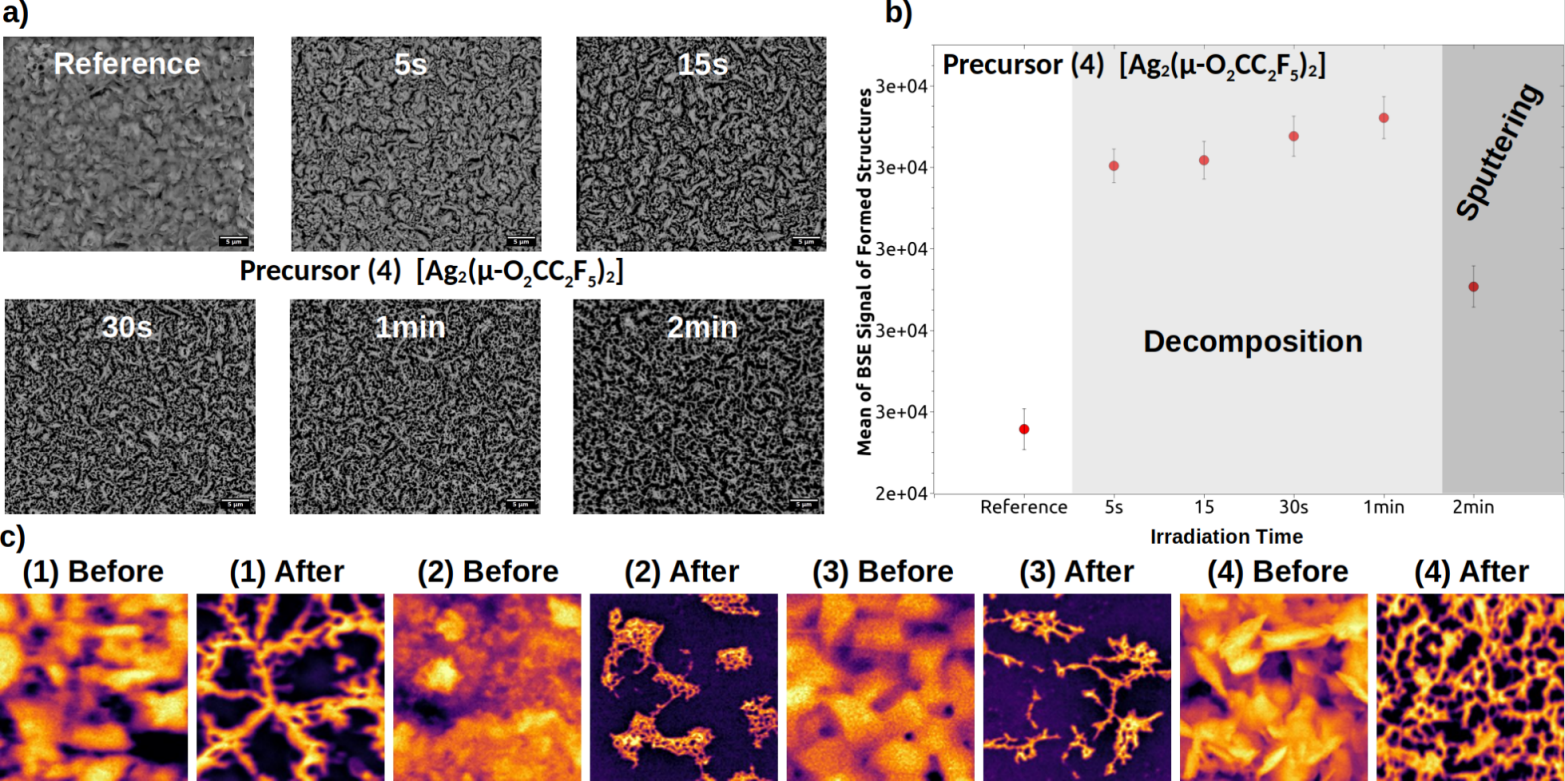
(a) SEM BSE morphology evolution studies of precursor **4** layer decomposition during gallium FIB irradiation experiments. (b) SEM BSE intensity (proportional to the atomic number *Z*) changes of the formed structures, as in (a), during gallium FIB irradiation experiments for precursor **4**. The BSE intensity increases during FIB irradiation (metal content increases), the precursor decomposes up to sputtering point at which the formed metal-rich structures do not further develop and sputtering of the structures by FIB gallium ions dominates. (c) SEM BSE morphology before (initial surface) and after gallium FIB decomposition experiments of the layers of precursors **1**–**4**.

In these experiments, a 30 keV energy beam was employed for raster scanning over a 50 µm × 50 µm square area with a dwell time of 200 ns. Ion beam current and duration of the experiments were adjusted (within a range of 1 to 10 nA for the ion current, and 10 s to 10 min the irradiation) in order to achieve the optimal ion fluence necessary for the decomposition of the entire precursor layer. Time-dependent changes in the morphology of the irradiated films were tracked using the SEM BSE signal, which is directly proportional to the average atomic number *Z*. BSE morphology changes during Ga FIB experiments for the precursor **4** are presented in Figure 2a. The initial layer consists of grain-like structures, the blurred BSE contrast indicates that the layer has a rather homogeneously distributed chemical composition, and only a few low-contrast grooves are visible. During ion irradiation, the BSE contrast increases strongly already in the very early stages of bombardment. An increasing number of (“grid”) dark contrast grooves separating elongated island-like features become more apparent. The precursor layer underwent decomposition leading to the development of surface features enriched with metallic element of the primary film.

The quantitative changes are presented in Figure 2b, which shows the mean BSE signal intensity, acquired while imaging the surface structures formed, as a function of the ion beam irradiation time. It is seen that, at the initial stages of irradiation, the BSE signal rapidly rises (in comparison to the not irradiated reference sample). Next one sees increase of the metal content, and the precursor decomposes. Finally, the BSE signal rapidly drops; all precursor material already decomposed into the metal-rich phase, the sputtering of the metal phase has begun to dominate, and the layer is getting thinner and thinner and finally is sputtered off (as in Figure 1b–e). From this dependence, one can determine the optimal sputtering point, that is, the maximal ion dose to decompose the given precursor layer without dominant sputtering, after which we stop the experiments. In Figure 2c, BSE morphologies of the four studied precursors **1**–**4** are shown. The BSE morphology of the initial (reference) material is presented together with the BSE morphology of the precursors after Ga FIB experiments regarding the optimal sputtering point.

**Figure 3:**
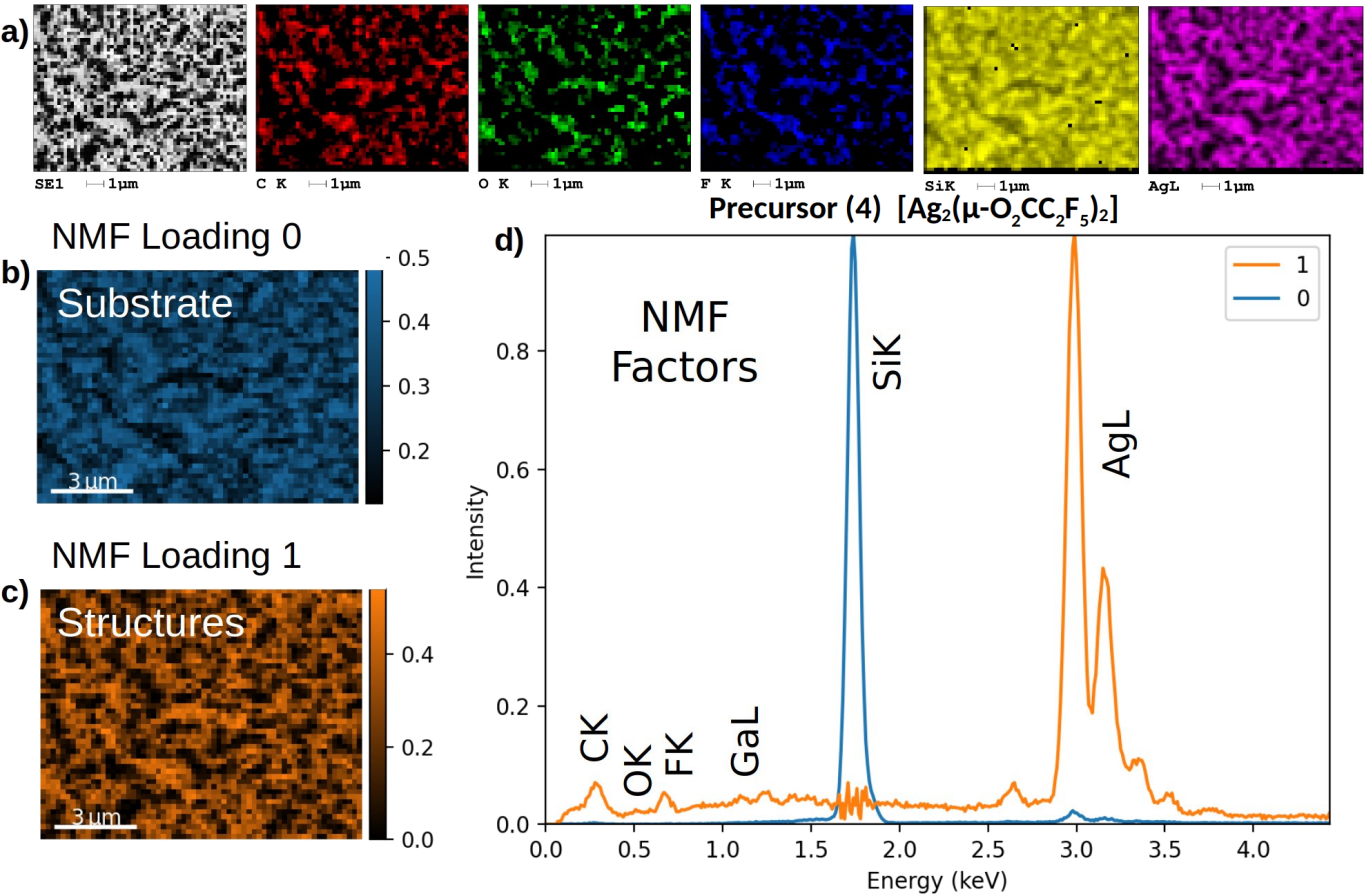
(a) Results of the SEM EDX hyperspectral mapping analysis of the precursor **4** layer after gallium FIB decomposition experiments; from left: BSE image and corresponding elemental net count maps of C K, O K, F K, Si K, and Ag L lines. Results of machine learning NMF decomposition of the collected SEM EDX hyperspectral data b)–d). NMF loadings showing spatial distribution of the NMF decomposition components b) substrate, c) structures together with NMF factors corresponding to the decomposed EDX signal d). It is seen that the EDX silicon substrate signal is succesfully separated from the signal of the metal-rich structures (Si K peak). This allows for the chemical composition quantification via EDX ZAF method.

It is seen that, for all precursors the morphology changed significantly; all precursors decomposed into a metal-rich phase. The initially compact films change to a network of interconnected and elongated island-like structures. In order to determine the chemical composition of the resulting structures, EDX data were collected in the hyperspectral mode, that is, for each *x*, *y*-position a full EDX spectrum was collected. The EDX measurements were performed at 20 keV electron beam energy. The EDX data were analyzed first by generating net count (background subtracted) maps of the elements. Figure 3a shows the SEM EDX hyperspectral mapping analysis results for precursor **4** after Ga FIB decomposition experiments, including the BSE image and the corresponding elemental net count Kα maps for the elements C, O, F, and Si, as well as the Ag Lα map (see Supporting Information File 1, Figure S2–S4 for other precursors). In all cases, the maps show that the formed structures are enriched in metal. In the next step, the EDX data were processed by machine learning NMF as described in details in Jany and colleagues [33]. In Figure 3b,c, the spatial distribution of the individual elements derived from NMF is depicted in the form of loading plots for the substrate and the structures. These plots are shown in color to visually distinguish between the different elements. The substrate layer is shown in blue, while the structure features are displayed in orange. Additionally, the NMF factors that correspond to the decomposed energy-dispersive X-ray spectra are shown in Figure 3d. It is evident that the performed NMF has successfully separated the EDX signal originating from the grown structures from the EDX signal coming from the silicon substrate. Subsequently, these distinct EDX signals were employed to quantify the chemical composition of the structures using an energy-dispersive X-ray EDX ZAF method in a standardless approach.

## Results and Discussion

The chemical compositions of the formed structures are presented in Table 1. The table shows the parameters of gallium ion FIB experiments carried out on deposited layers of **1**–**4** at their optimal sputtering point, along with SEM EDX chemical composition analysis of the formed structures on the sample surface. For the initial precursor composition please see Table S2 in Supporting Information File 1. To ensure a fair and accurate comparison between different precursor parameters, it was necessary to take into account the different thicknesses of the precursor layers. In order to achieve this, we decided to utilize an ion fluence that had been normalized to the specific height of the individual precursor layer, *Fh* = fluence/(layer height). The fluence [ions·cm^−2^] and fluence per height [ions·cm^−2^·μm^−1^] values are also presented in Table 1 as well as the results from previous FEBID experiments for the complexes **3** and **4** [5,15,23]. In the absence of FIBID data for the precursors, the FEBID experiments provide a valuable means of comparison to the gas-phase studies. The results allow us to evaluate the performance of the new precursors by correlating ion beam parameters with the chemical composition of the forming structures. Analysis of the data in Table 1 enables us to determine how the potential new precursor is modified under the Ga FIB ion beam exposure. It is seen that for the precursor **3** and **4**, the final metal content obtained under Ga^+^ FIB irradiation is comparable to that obtained in FEBID experiments.

**Table 1:** Fluence and *Fh* = Fluence/(layer height) of the final Ga FIB experiments performed (at optimal sputtering point) on layers of precursors **1**–**4**, together with SEM EDX chemical composition of the formed structures after precursor decomposition. The volume/dose is also estimated for each precursor. Results of previously performed FEBID experiments are given for comparison. In the final column, a metric called precursor score (*Sp*) is included, which is calculated as metal content divided by [gallium content × log(*Fh*)].

Precursor	Parameters of Ga FIB experiments			SEM EDX content of the formed metal-rich structures on the sample surface [atom %]						*Sp*
	fluence [ions/cm^2^]	*Fh* [ions·cm^−2^·μm^−1^]	volume/dose [μm^3^·nC^−1^]	C	N	O	F	Ga	metal	

**1**	1.2 × 10^18^	1.47 × 10^17^	0.0090 (0.0054)	62.57 (0.13)	–	7.73 (1.5)	–	22.22 (0.44)	Cu: 15.39 (0.31)	0.040
**2**	4.49 × 10^16^	7.45 × 10^15^	0.023 (0.014)	24.5 (4.9)	11.6 (2.3)	6.65 (1.3)	24.2 (4.8)	<0.3	Cu: 33.1 (0.66)	6.951
**3**	1.5 × 10^17^	1.06 × 10^17^	0.0030 (0.0018)	22.21 (0.89)	–	10.98 (2.2)	15.18 (0.61)	8.69 (0.35)	Cu: 42.93 (0.86)	0.290
**3** FEBID [15]	–	–	–	51–5	–	2–44	44–8	–	Cu: 19–23	–
**4**	1.5 × 10^16^	1.40 × 10^16^	0.21 (0.13)	17.27 (1.7)	–	2.99 (1.5)	15.74 (1.6)	1.44(0.72)	Ag: 64.0 (1.3)	2.753
**4** FEBID [23]	–	–	–	20–47	–	1–34	3–5	–	Ag: 33–76	–
Pt FIBID [35–36]	–	–	0.5	24–58	–	2–4	–	20-28	Pt: 24–46	–

**Figure 4:**
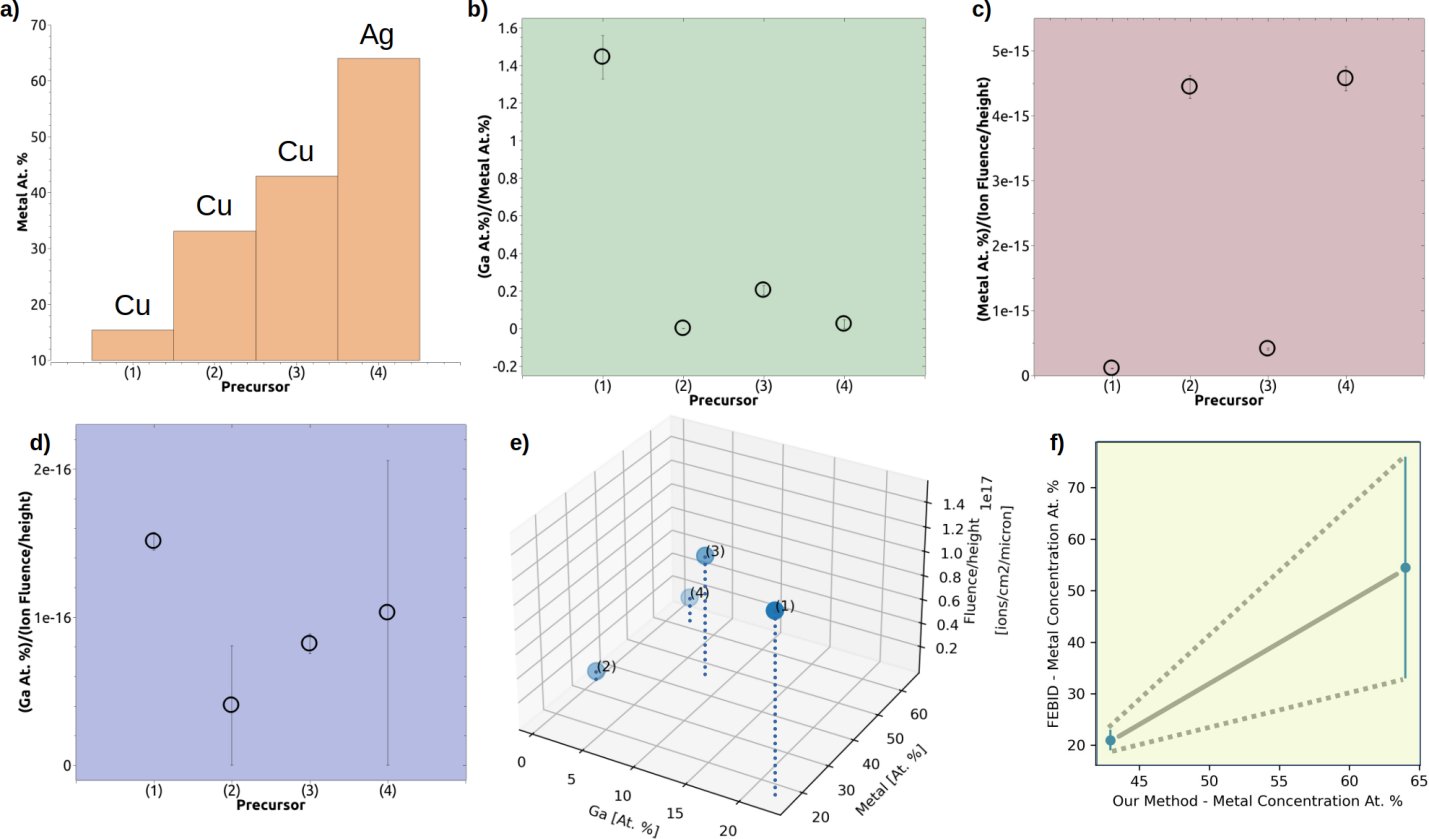
Investigation of chemical composition (precursor performance) for the studied precursors **1**–**4** after gallium FIB experiments. (a) Metal content in atom % in the final structures. It is seen that precursor **4** yields the highest metal content. (b) Ratio of gallium to metal in the final structures. Precursor **1** accumulates the highest amount of gallium in comparison to metal. Ratio of (c) metal and (d) gallium atom % to ion fluence *Fh*. Precursors **2** and **4** decompose most easily during FIB experiments producing the highest amount of metal per ion fluence *Fh*. Precursor **1** absorbs the most gallium in terms of amount of gallium per fluence during irradiation. (e) Three-dimensional representation showing the relationship between gallium content, metal content, and ion fluence *Fh*. (f) Average metal content in atomic percent obtained for precursors **3** and **4** using FEBID versus the metal content of the precursors as determined by our layer decomposition method.

It can be seen that the gallium content increases with *Fh*. It is also worth to notice that the 30 keV gallium ion range in copper and silver is almost the same (the longitudinal ranges of gallium ions as calculated by SRIM are 11.6 nm for copper, 11.0 nm for silver, and 28.6 nm for silicon). Additionally, we calculated the volume-to-dose rate for each studied complex based on the dimensions of the final structures and the applied ion dose. This enables us to compare the ability of studied precursors to form metal-rich structures under gallium ion FIB irradiation with that of the commonly used Pt precursor trimethyl(methylcyclopentadienyl)platinum(IV) [Pt(η^5^-CpMe)Me_3_] [35–36]. The results indicate that precursors **2** and **4** exhibit a performance comparable to that of the Pt precursor, with precursor **4** being the most comparable. Our ultimate goal was to identify the promising precursor that would exhibit a high metal content while minimizing gallium accumulation and decomposing efficiently at low ion fluence *Fh*. Detailed analyses of the chemical composition of the formed structures are presented in Figure 4. It is evident that the precursor **4** gave the highest metal content among the tested compounds, as observed in the atomic percentage values in Figure 4a. Figure 4b presents a visual representation of the ratio of gallium to the total metal content in the final structures. This allows for a comparison of gallium content to other metals within these structures, highlighting variations or trends among the different precursors. The data shows that, in the final metallic structures, precursor **1** yields a significantly higher gallium-to-other-metals ratio compared the other precursors. We also examined the relationship between ion fluence *Fh* and both the total metal content and the gallium content in the resulting structures. This analysis is illustrated in Figure 4c and Figure 4d, which show the metal and gallium atomic percentages normalized to *Fh*. The analysis revealed that precursors **2** and **4** yielded the highest metal content per unit of *Fh*, indicating that they decomposed most efficiently under Ga ion irradiation. In contrast, precursor **1** exhibited a higher propensity for gallium absorption during irradiation. By analyzing the ion beam-induced decomposition of the precursors, which resulted in the formation of structures with varying chemical composition, we were able to assign performance scores to each precursor. To visualize the relationship between the three key parameters, that is, gallium content, metal content, and *Fh*, we presented them in a three-dimensional scatter plot (Figure 4e). To support the selection of the most suitable precursor, we developed a precursor score *Sp* = (metal content)/(galium content × log(*Fh*)), as summarized in Table 1. This scoring system prioritizes precursors that, upon decomposition, yield structures with high metal content and low gallium content at the minimal ion fluence *Fh* and accurately reflects each precursor’s overall performance. This parameter allowed for a quick numerical assessment of precursor performance, helping to identify the most suitable candidates for further investigation or use in FIBID. In this context, the results indicate that precursors **2** and **4** appeared to yield the best balance among all three optimized parameters and among the four studied compounds. Finally, to validate our approach, we compared the FEBID metal content data available in the literature (Table 1) with the values obtained using the present layer decomposition methodology (Figure 4f). The figure shows the average atomic metal content for precursors **3** and **4** measured in FEBID experiments versus the metal content determined using our ion-beam-based decomposition method. It is seen that, on average, there is a relation between these two values of the metal content. Independently of the different mechanisms governing layer decomposition under ion beam irradiation and gas-phase deposition in FEBID, our results demonstrate that the proposed method provides valuable insight into the behavior of metal deposition from metal-organic precursors. We also believe that our methodology could be used as a valuable tool for rapid precursor screening and evaluation.

All of our precursor tests, as well as the final precursor scoring process, were carried out using a straightforward and widely accessible testing method. This approach involved conducting precursor layer tests on SEM and utilizing both BSE and EDX analyses. By employing this commonly available methodology, we aimed to ensure that our results would be replicable and relevant to a wide range of potential precursors. The collected SEM BSE and EDX data together with an exemplary Python Jupyter notebook to analyze EDX hyperspectral data are freely available from Zenodo [37].

## Conclusion

In this research, we studied the ion-beam-induced decomposition of four Cu or Ag metal-organic precursors (i.e., ([Cu_2_(μ-O_2_C*t*-Bu)_4_] (**1**), [Cu_2_(NH_2_(NH=)CC_2_F_5_)_2_(µ-O_2_CC_2_F_5_)_4_] (**2**), [Cu_2_(µ-O_2_CC_2_F_5_)_4_] (**3**), and [Ag_2_(μ-O_2_CC_2_F_5_)_2_] (**4**)) exposed to 30 keV gallium FIB irradiation. Individual precursor layers were deposited onto silicon substrates via sublimation and subsequently exposed to gallium FIB irradiation. The optimal ion fluence for each precursor was determined by monitoring changes in the BSE signal intensity associated with the evolving surface morphology. The resulting metal-rich surface structures were analyzed using SEM EDX processed by machine learning techniques to extract the chemical composition. The study revealed that the silver precursor **4** produced the highest overall metal content in the final structures, while the copper precursor **1** resulted in the highest level of gallium incorporation. The copper precursors **2** and **4** demonstrated superior performance in terms of metal yield per unit of ion fluence per height, *Fh*, indicating that they decomposed more readily compared to the other tested precursors. To evaluate the overall effectiveness of each precursor, a scoring system called precursor score, *Sp*, was introduced, which incorporates metal content, gallium content, and ion fluence per height required for decomposition. The results showed that precursors **2** and **4** achieved the highest precursor scores, indicating their superior ability to balance all three parameters. It is worth to notice that precursor **4**, which is Ag-based, was also tested in FEBID and gave very good results. To validate our approach, we compared our results with FEBID data, revealing a consistent relationship between the two methods regarding the final metal content. The study highlights the importance of understanding the chemical composition of various potential precursors for producing metal-rich structures using Ga FIB techniques. By employing a straightforward testing methodology, we identified promising carboxylate complexes that could potentially be applied across various fields and applications. Analyzing precursor layer decomposition presents unique challenges compared to gas-phase metal deposition due to differing underlying processes. However, our method consistently provides valuable insights into the physics governing metal deposition from metal-organic precursors. This approach shows significant potential as a tool for evaluating and selecting promising precursor materials and could accelerate the development of metal-organic precursors specifically tailored for FIB, offering a cost-effective route to novel nanofabrication applications.

## Supporting Information

File 1 Additional data.

## Data Availability

The collected experimental data are available from Zenodo https://doi.org/10.5281/zenodo.11354527

## References

[1] Li P, Chen S, Dai H, Yang Z, Chen Z, Wang Y, Chen Y, Peng W, Shan W, Duan H (2021). Nanoscale.

[2] Barth S, Huth M, Jungwirth F (2020). J Mater Chem C.

[3] Bruchhaus L, Mazarov P, Bischoff L, Gierak J, Wieck A D, Hövel H (2017). Appl Phys Rev.

[4] De Teresa J M, Orús P, Córdoba R, Philipp P (2019). Micromachines.

[5] Utke I, Swiderek P, Höflich K, Madajska K, Jurczyk J, Martinović P, Szymańska I B (2022). Coord Chem Rev.

[6] Fang C, Chai Q, Lin X, Xing Y, Zhou Z (2021). Mater Des.

[7] Córdoba Castillo R (2014). Ferromagnetic Iron Nanostructures Grown by Focused Electron Beam Induced Deposition. Functional Nanostructures Fabricated by Focused Electron/Ion Beam Induced Deposition.

[8] Matsui S, Kaito T, Fujita J, Komuro M, Kanda K, Haruyama Y (2000). J Vac Sci Technol, B: Microelectron Nanometer Struct–Process, Meas, Phenom.

[9] Huang Y, Yin K, Li B, Zheng A, Wu B, Sun L, Nie M (2024). Nanoscale Horiz.

[10] Sychugov I, Nakayama Y, Mitsuishi K (2009). J Phys Chem C.

[11] Allen F I (2021). Beilstein J Nanotechnol.

[12] Utke I, Michler J, Winkler R, Plank H (2020). Micromachines.

[13] Indrajith S, Rousseau P, Huber B A, Nicolafrancesco C, Domaracka A, Grygoryeva K, Nag P, Sedmidubská B, Fedor J, Kočišek J (2019). J Phys Chem C.

[14] Höflich K, Hobler G, Allen F I, Wirtz T, Rius G, McElwee-White L, Krasheninnikov A V, Schmidt M, Utke I, Klingner N (2023). Appl Phys Rev.

[15] Berger L, Jurczyk J, Madajska K, Edwards T E J, Szymańska I, Hoffmann P, Utke I (2020). ACS Appl Electron Mater.

[16] Córdoba R, Ibarra A, Mailly D, Guillamón I, Suderow H, De Teresa J M (2020). Beilstein J Nanotechnol.

[17] Manoccio M, Esposito M, Passaseo A, Cuscunà M, Tasco V (2021). Micromachines.

[18] Lee J S, Hill R T, Chilkoti A, Murphy W L (2020). Biomater Sci.

[19] Nakagawa Y, Yamaoka T, Sato M, Yamamoto M, Glanville J (1988). Proc SPIE.

[20] Utke I, Hoffmann P, Melngailis J (2008). J Vac Sci Technol, B: Microelectron Nanometer Struct–Process, Meas, Phenom.

[21] Jungwirth F, Porrati F, Knez D, Sistani M, Plank H, Huth M, Barth S (2022). ACS Appl Nano Mater.

[22] Martinović P, Rohdenburg M, Butrymowicz A, Sarigül S, Huth P, Denecke R, Szymańska I B, Swiderek P (2022). Nanomaterials.

[23] Berger L, Madajska K, Szymanska I B, Höflich K, Polyakov M N, Jurczyk J, Guerra-Nuñez C, Utke I (2018). Beilstein J Nanotechnol.

[24] Höflich K, Jurczyk J, Zhang Y, Puydinger dos Santos M V, Götz M, Guerra-Nuñez C, Best J P, Kapusta C, Utke I (2017). ACS Appl Mater Interfaces.

[25] Jurczyk J, Höflich K, Madajska K, Berger L, Brockhuis L, Edwards T E J, Kapusta C, Szymańska I B, Utke I (2023). Nanomaterials.

[26] Höflich K, Jurczyk J M, Madajska K, Götz M, Berger L, Guerra-Nuñez C, Haverkamp C, Szymanska I, Utke I (2018). Beilstein J Nanotechnol.

[27] Tripathi S K, Shukla N, Kulkarni V N (2008). Nucl Instrum Methods Phys Res, Sect B.

[28] Guan X, Yan R (2020). Synlett.

[29] Butrymowicz-Kubiak A, Luba W, Madajska K, Muzioł T, Szymańska I B (2024). New J Chem.

[30] Szłyk E, Szymańska I (1999). Polyhedron.

[31] Szłyk E, Łakomska I, Grodzicki A (1993). Thermochim Acta.

[32] Madajska K, Szymańska I B (2021). Materials.

[33] Jany B R, Janas A, Krok F (2017). Nano Lett.

[34] Madajska K (2022).

[35] Tao T, Ro J, Melngailis J, Xue Z, Kaesz H (1990). J Vac Sci Technol, B: Microelectron Process Phenom.

[36] Puretz J, Swanson L W (1992). J Vac Sci Technol, B: Microelectron Nanometer Struct–Process, Meas, Phenom.

[R37] 37Jany, B. R. Data for "Evaluating Metal-Organic Precursors for Focused Ion Beam Induced Deposition through Solid-Layer Decomposition Analysis", Zenodo 2024. doi:10.5281/zenodo.11354527

