# Supplementary material for: Evaluating metal-organic precursors for focused ion beam-induced deposition through solid-layer decomposition analysis
Source: Beilstein J Nanotechnol. 2025 Nov 4;16:1942–51. doi: 10.3762/bjnano.16.135 (PMC12599390; doi:10.3762/bjnano.16.135)
Supplement: File 1 — Additional data. [file Beilstein_J_Nanotechnol-16-1942-s001.pdf]

BEILSTEIN JOURNAL OF NANOTECHNOLOGY

Supporting Information

for

Evaluating metal-organic precursors for focused ion beam-induced deposition through solid-layer decomposition analysis

Benedykt R. Jany, Katarzyna Madajska, Aleksandra Butrymowicz-Kubiak, Franciszek Krok and Iwona B. Szymańska

Beilstein J. Nanotechnol. **2025,** *16,* 1942–1951. doi:10.3762/bjnano.16.135

Additional data

License and Terms: This is a supporting information file under the terms of the Creative Commons Attribution License (https://creativecommons.org/licenses/by/4.0). Please note that the reuse, redistribution and reproduction in particular requires that the author(s) and source are credited and that individual graphics may be subject to special legal provisions.
The license is subject to the *Beilstein Journal of Nanotechnology* terms and conditions: (https://www.beilstein-journals.org/bjnano/terms)

Materials and precursors synthesis

Pivalic acid (99%), $CuCO_3 \cdot Cu(OH)_2$ (>95%), anhydrous acetonitrile (99.8%), and C_2F_5COOH (97%) were purchased from Merck (Saint Louis, MO, USA), absolute ethanol (≥99.8%) – from Honeywell (Charlotte, USA), sodium hydroxide (p.a) – from Avantor (Avantor Performance Materials, Poland S.A.), copper(II) nitrate trihydrate (99%) and $AgNO_3$ (99,9%) – from Chempur (Poland). Pentafluoropropylamidine $C_2F_5C(=NH)NH_2$ (AMDH) (98.7%) was from Apollo Scientific (Stockport, UK). Copper(II) pivalate $[Cu_2(\mu_3\text{-}O_2CtBu)_2(\mu_2\text{-}O_2CtBu)_2]_n$ (for simplicity, we use a formula $[Cu_2(\mu\text{-}O_2CtBu)_4]_n$[1] (**1**)), copper(II) pentafluoropropionate $[Cu_2(\mu\text{-}O_2CC_2F_5)_4]$[2] (**3**), and silver(I) pentafluoropropionate $[Ag_2(\mu–O_2C_2F_5)_2]$[3] (**4**) were prepared as earlier reported. Based on the synthesis developed by us, the copper(II) amidine−carboxylate complex $[Cu_2(NH_2(NH=)CC_2F_5)_2(\mu–O_2CC_2F_5)_4]$[4] (**2**) was synthetized. The Si(111) substrates were purchased from the Institute of Microelectronics and Photonics, Center for Electronic Materials Technology in Warsaw (Lukasiewicz Research Network, Poland).

Infrared spectra analysis of the original complexes $[Cu_2(\mu\text{-}O_2CtBu)_4]_n$ (1)[5], $[Cu_2(NH_2(NH=)CC_2F_5)_2(\mu–O_2CC_2F_5)_4]$ (2)[6], $[Cu_2(\mu\text{-}O_2CC_2F_5)_4]$ (3), and $[Ag_2(\mu–O_2C_2F_5)_2]$ (4)

Table S1: Selected characteristic IR absorption bands (cm^{-1}) for $[Cu_2(\mu\text{-}O_2CtBu)_4]_n$ (1), $[Cu_2(NH_2(NH=)CC_2F_5)_2(\mu–O_2CC_2F_5)_4]$ (2), $[Cu_2(\mu\text{-}O_2CC_2F_5)_4]$ (3), and $[Ag_2(\mu–O_2C_2F_5)_2]$ (4).

Vibrations	(**1**)	(**2**)	(**3**)	(**4**)
$\nu_{as}COO$	1578 and 1530	1657	1640	1603
$\nu_{s}COO$	1412	1418	1421	1423
$\nu_{as}NH_2$	−	3390	−	−
ν(=NH)	−	3240	−	−
δNH_2	−	1603	−	−
νN=C−N	−	1510	−	−

1 X. Guan and R. Yan, Copper-Catalyzed Synthesis of Alkyl-Substituted Pyrrolo[1,2-*a*]quinoxalines from 2-(1*H*-Pyrrol-1-yl)anilines and Alkylboronic Acids, *Synlett.*, 2020, **31**, 359–362.

2 Szłyk, E.; Szymańska, I. Studies of new volatile copper(I) complexes with triphenylphosphite and perfluorinated carboxylates. Polyhedron 1999, 18, 2941–2948.

3 E. Szłyk, I. Łakomska, A. Grodzicki, Thermal and spectroscopic studies of the Ag(I) salts with fluorinated carboxylic and sulfonic acid residues, Thermochim. Acta. 223 (1993) 207–212, https://doi.org/10.1016/0040-6031(93)80136-X.

4 Madajska, K.; Szymańska, I.B. New volatile perfluorinated amidine–carboxylate copper(II) complexes as promising precursors in CVD and FEBID methods. Materials (Basel). 2021, 14, 3145, doi:10.3390/ma14123145.

5 A. Butrymowicz-Kubiak, W. Luba, K. Madajska, T. Muzioł and I. B. Szymańska, Pivalate complexes of copper(II) with aliphatic amines as potential precursors for depositing nanomaterials from the gas phase, New J. Chem., 2024, 48, 6232

6 Madajska, K.; Szymańska, I.B. New volatile perfluorinated amidine–carboxylate copper(II) complexes as promising precursors in CVD and FEBID methods. Materials (Basel). 2021, 14, 3145, doi:10.3390/ma14123145.

Table S2: Initial atomic composition of the precursor layer.

Precursor	Atomic %					
	Cu/Ag	C	H	N	F	O
(1) $[Cu_2(\mu\text{-}O_2C\textit{t}Bu)_4]_n$	3.03	30.30	54.55	0.00	0.00	12.12
(2) $[Cu_2(NH_2(NH{=})CC_2F_5)_2(\mu\text{-}O_2CC_2F_5)_4]$	2.94	26.47	5.88	8.82	44.12	11.76
(3) $[Cu_2(\mu\text{-}O_2CC_2F_5)_4]$	4.76	28.57	0.00	0.00	47.62	19.05
(4) $[Ag_2(\mu\text{-}O_2C_2F_5)_2]$	9.09	27.27	0.00	0.00	45.45	18.18

SEM BSE Image Intensity Analysis

The image analysis, as depicted in Fig. S1, employed a rigorous approach to extract the brightness signal (backscattered electron intensity, BSE) from the metal-rich nanostructures. The initial step involved thresholding using ImageJ/FIJI's default method to effectively separate the metal-rich structures from the underlying silicon substrate. This segmentation enabled the isolation of the regions of interest, thereby facilitating the subsequent analysis.

Subsequently, a histogram of BSE intensities was generated by selectively marking the red areas in the image, which corresponded to the metal-rich nanostructures. This process allowed for the compilation of a comprehensive distribution of BSE intensity values specific to these structures. The mean BSE intensity was subsequently calculated from this data, providing qualitative information about the metal content in the structures since the BSE intensity is proportional to atomic number Z.

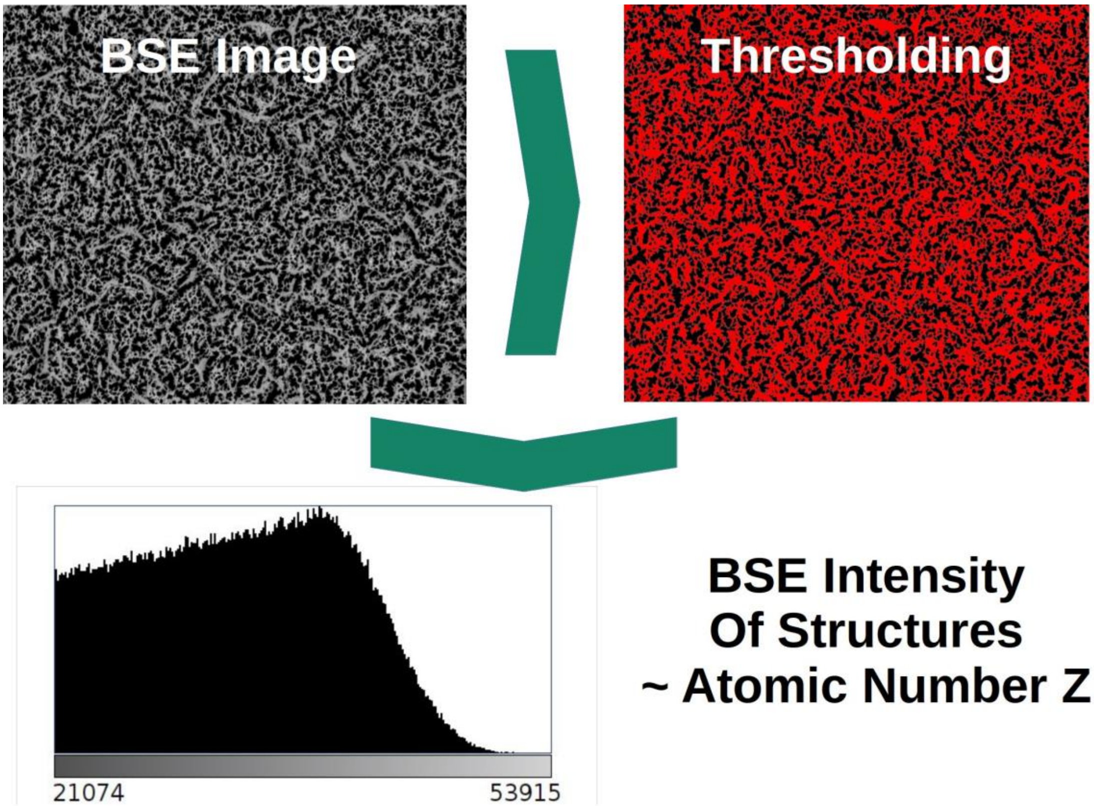

Figure S1: A comprehensive diagram illustrates the step-by-step workflow for analyzing the brightness signal (backscattered electron intensity, BSE) in images of metal-rich structures obtained after focused ion beam (FIB) irradiation of precursor materials. This schematic representation provides a clear visual overview of the methods employed to extract meaningful information from the BSE images.

SEM EDX analysis of the metal rich structures after precursors (1-4) FIB irradiation

Precursor (1) after FIB Irradiation

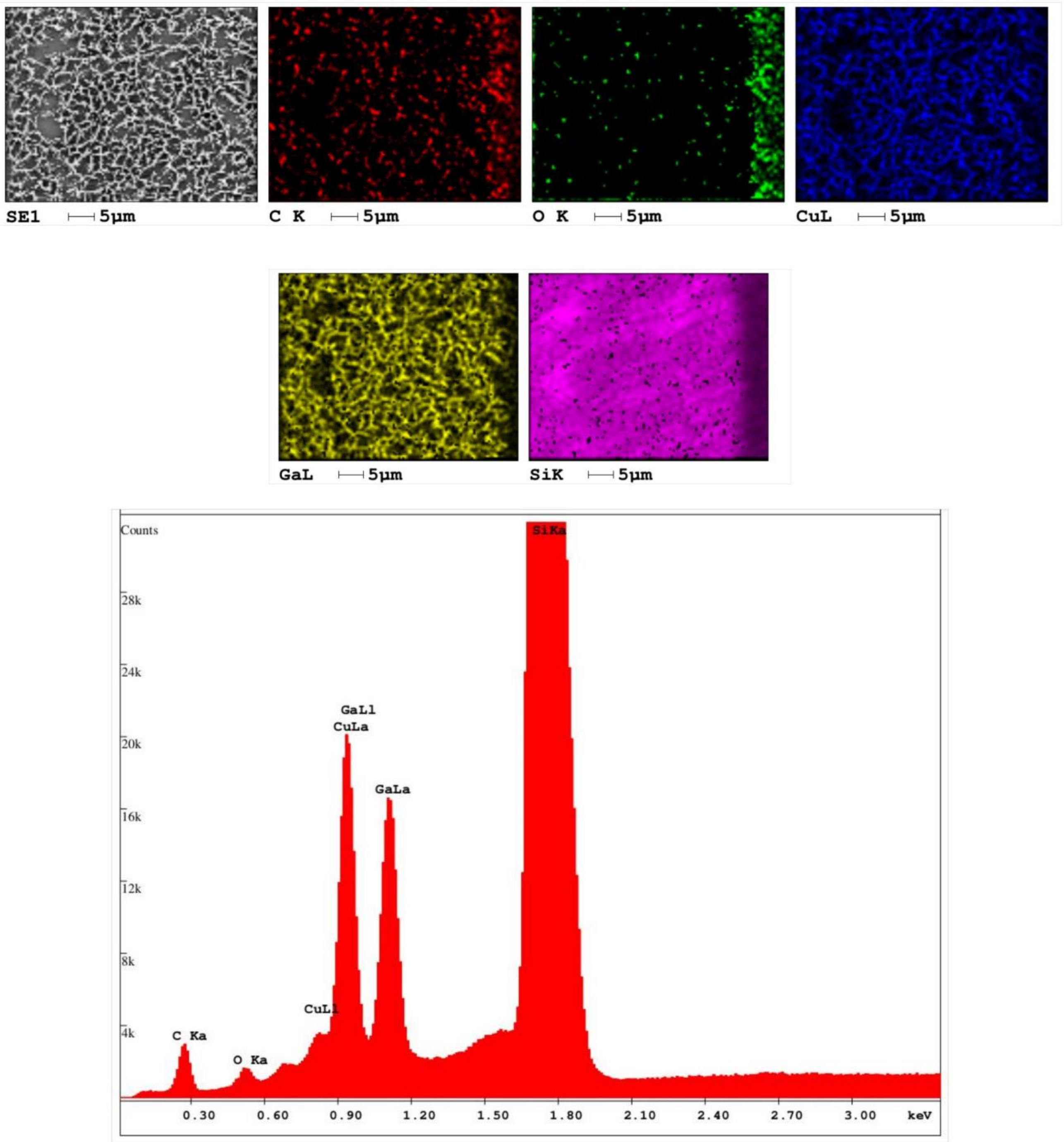

Figure S2: SEM EDX analysis of the precursor layer (1) [Cu_2(μ-O_2C*t*Bu)$_4$]*n* after FIB irradiation has yielded a set of high-resolution background-subtracted SEM EDX netto-count maps and a comprehensive cumulative EDX spectrum. These maps provide detailed visualizations of the elemental composition and spatial distribution of the sample, while the cumulative spectrum offers quantitative information on the relative abundance of each element present in the material.

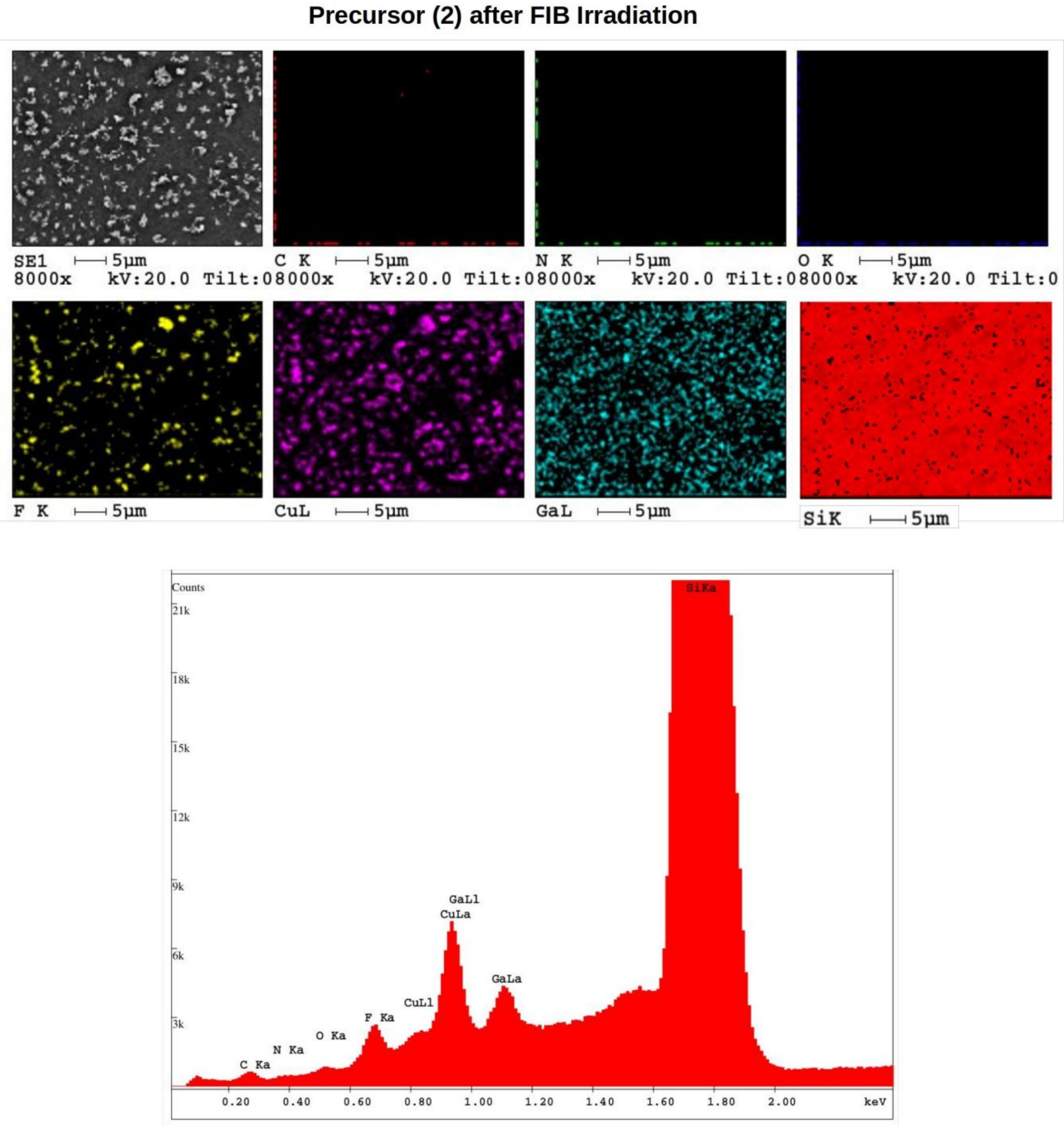

Figure S3: SEM EDX analysis of the precursor layer (2) [$Cu_2(NH_2(NH=)CC_2F_5)_2(\mu\text{-}O_2CC_2F_5)_4$] after FIB irradiation has yielded a set of high-resolution background-subtracted SEM EDX netto-count maps and a comprehensive cumulative EDX spectrum. These maps provide detailed visualizations of the elemental composition and spatial distribution of the sample, while the cumulative spectrum offers quantitative information on the relative abundance of each element present in the material.

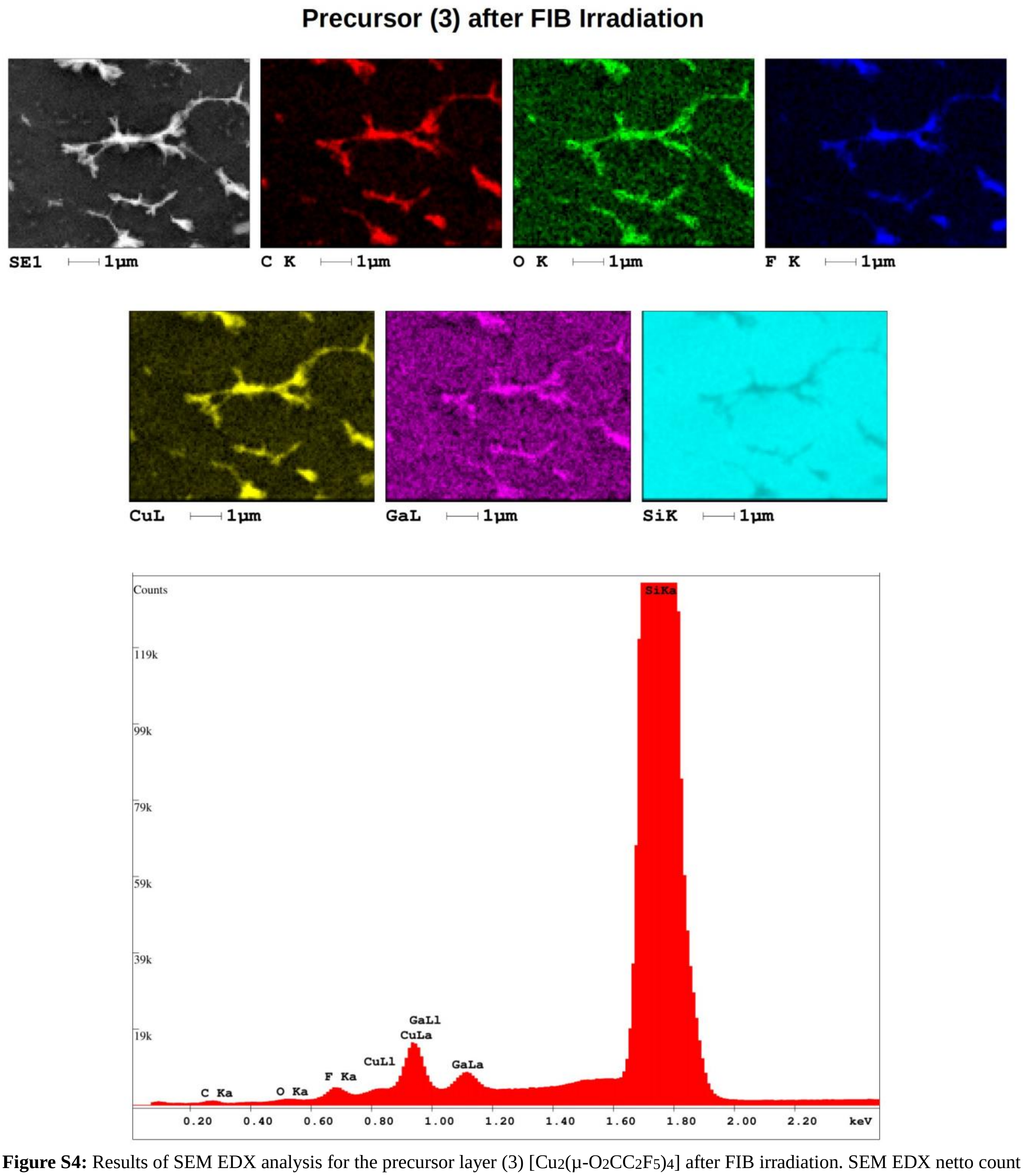

Figure S4: Results of SEM EDX analysis for the precursor layer (3) $[Cu_2(\mu\text{-}O_2CC_2F_5)_4]$ after FIB irradiation. SEM EDX netto count maps (background subtracted) and corresponding cumulative EDX spectrum. SEM EDX analysis of the precursor material (1) after FIB irradiation has yielded a set of high-resolution background-subtracted SEM EDX net-to-count maps and a comprehensive cumulative EDX spectrum. These maps provide detailed visualizations of the elemental composition and spatial distribution of the sample, while the cumulative spectrum offers quantitative information on the relative abundance of each element present in the material.

SEM Electron Beam Irradiation

Following the FIB irradiation of the precursor layer, we also conducted electron beam irradiation experiments under identical conditions and parameters as those employed for ion beam irradiation. Fig. S5 presents SEM BSE images before and after electron beam irradiation, providing a visual comparison of the effects of these two forms of radiation on the precursor layer. This highlights the distinct effects of electron and ion beams on the precursor layer, revealing differences in their transformations.

Notably, despite identical parameters used in this experiment and previous FIB ion beam experiments, the electron beam irradiation does not lead to the decomposition of the precursor layer, differing from the layer transformations observed under FIB ion beam conditions.

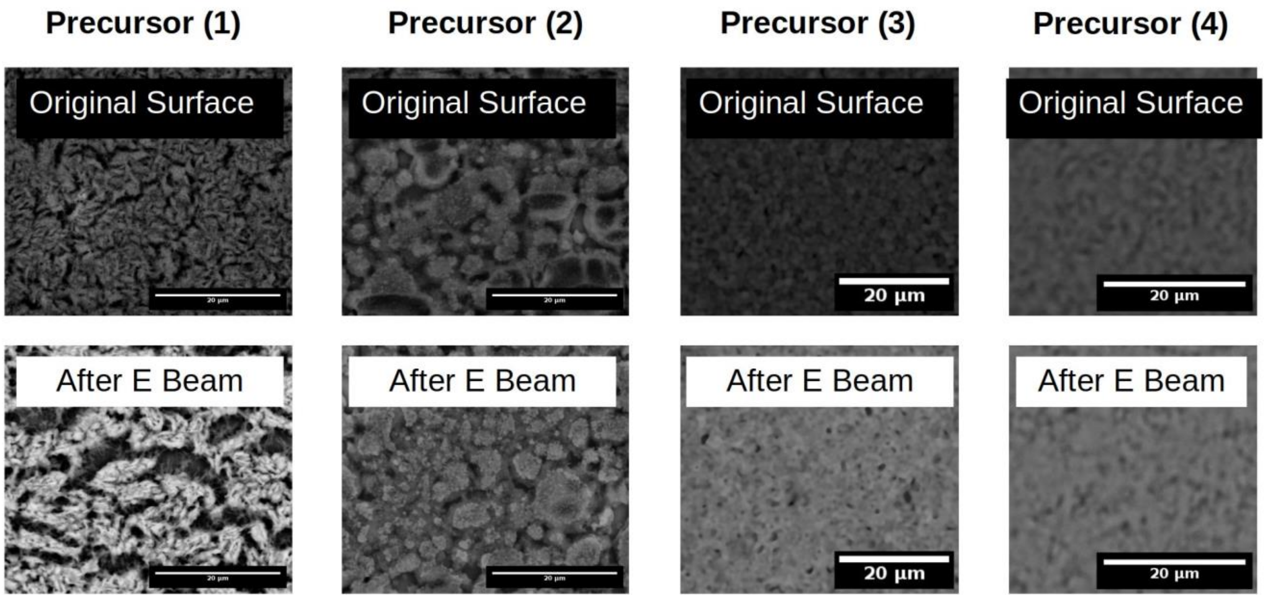

Figure S5: SEM BSE images are provided for comparison, featuring the original surface morphology of the precursor layers as deposited (upper row) alongside that after electron beam irradiation (lower row). This comparison highlights the distinct effects of these two forms of radiation on the precursor material. Notably, despite identical parameters used in this experiment and previous FIB ion beam experiments, the electron beam irradiation does not lead to decomposition of the precursor layer, differing from the layer transformations observed under FIB ion beam conditions.
